# Adverse events after the transjugular intrahepatic portal shunt are linked to serum metabolomic changes following the procedure

**DOI:** 10.3389/fmolb.2023.1168782

**Published:** 2023-05-15

**Authors:** Quan Chen, Li Bao, Zhendong Yue, Lei Wang, Zhenhua Fan, Fuquan Liu

**Affiliations:** ^1^ Department of Interventional Therapy, Beijing Shijitan Hospital, Capital Medical University, Beijing, China; ^2^ Department of Pharmacy, Beijing Shijitan Hospital, Capital Medical University, Beijing, China

**Keywords:** transjugular intrahepatic portosystemic shunt, portal hypertension, liver function decline, metabolome, biomarker

## Abstract

**Background and Objective:** Transjugular intrahepatic portal shunt (TIPS) insertion could promote weight gain and muscle and fat mass increase in patients with cirrhosis. However, few studies have focused on metabolic changes after TIPS. This study aims to explore metabolic changes after TIPS and potential biomarkers of adverse events.

**Methods:** Peripheral and portal serum samples were collected before and after TIPS insertion. Untargeted metabolomics was performed using ultra-high-performance liquid chromatography-mass spectrometry. Spearman’s correlation analysis was used to determine the relationship between metabolites and clinical parameters. Metabolite set enrichment analysis was performed to explore enriched pathways. The predictive value of the metabolites was calculated by receiver operating characteristic curve (ROC) analysis.

**Results:** Metabolites in the peripheral and portal serum significantly changed early after TIPS. Some lipid metabolites were significantly correlated with liver function parameters. Both elevated and depleted metabolites were mainly enriched in amino acid metabolism. Nine and 12 portal metabolites have moderate predictive value in post-TIPS liver function decline and hepatic encephalopathy (HE), separately (area under curve >0.7).

**Conclusion:** Metabolites in the peripheral and portal veins significantly changed after TIPS. Some metabolic changes might be ascribed to liver function decline early after TIPS. Nine and 12 portal metabolites might be potential biomarkers in prediction of liver function decline and HE, separately.

## 1 Introduction

Portal hypertension is a common clinical syndrome frequently accompanied by ascites, varices, and bleeding, which is the leading cause of death and liver transplantation in patients with chronic liver disease (Tsochatzis et al., 2014; Simonetto et al., 2019). Transjugular intrahepatic portal shunt (TIPS) is an effective treatment for portal hypertension (García-Pagán et al., 2010; Bureau et al., 2017). Besides decreasing the portal pressure gradient, TIPS insertion could produce weight gain in malnourished patients with portal hypertension, as reported by previous studies (Trotter et al., 1998; Plauth et al., 2004). Weight gain after TIPS insertion usually increases muscle and fat mass (Dasarathy et al., 2011; Jahangiri et al., 2019; Artru et al., 2020). Importantly, sarcopenia, a surrogate marker of the malnourishment state, is independently associated with adverse outcomes in patients with cirrhosis (Tandon et al., 2021; Liu et al., 2022). Thus, the improvement of sarcopenia after TIPS is a favorable factor in the prognosis of patients with cirrhosis. In addition, weight gain following TIPS insertion was associated with beneficial changes in circulating adipokine levels, indicating that post-TIPS metabolism shifts to the anabolic function of fat mass (Thomsen et al., 2012). These studies showed that metabolism after TIPS significantly changed.

Metabolism is downstream of the gene regulatory and proteomic networks, reflecting life activities (Judge and Dodd, 2020). Thus, the comprehensive analysis of metabolites provides a more direct characterization label and a more sensitive disease detection method. Liquid chromatography-mass spectrometry (LC-MS)-based metabolomics helps explore novel circulating metabolomic profiles for many diseases (Wang et al., 2013; Stolzenberg-Solomon et al., 2020). An increasing number of studies have used LC-MS to explore potential predictors of prognosis from plasma, serum, urine, or tissue (Fan et al., 2016; Luo et al., 2018; Chen et al., 2022). In patients with advanced cirrhosis, lipids are generally suppressed, and sphingomyelins and cholesteryl esters can distinguish patients with different severity of cirrhosis (Claria et al., 2021). Furthermore, changes in polyunsaturated fatty acids and lipid metabolism are involved in the pathogenesis of lipid metabolism disorders in hepatitis C patients (Miyoshi et al., 2011). Additionally, cirrhotic patients with sarcopenia also had specific metabolic alternation. For example, arabinose, succinate, xylose, alpha galactose, hypoxanthine, formate, dimethylamine, isoleucine, and ethanol were the most represented in sarcopenic cirrhotic patients (Ponziani et al., 2021). However, few studies focused on the metabolic changes after TIPS and the impact of sarcopenia improvement.

As the amelioration of malnutrition after TIPS might contribute to a better prognosis for patients with cirrhosis, we hypothesize that analysis of metabolomic profile changes after TIPS might identify potential predictors of prognosis. Thus, we collected pre- and post-TIPS serum samples and performed detailed metabolomics analysis using ultra-high-performance liquid chromatography-mass spectrometry (UHPLC-QTOF/MS). This study aims to describe the metabolic change after TIPS and identify the specific metabolomic profiles in patients with significant liver function decline and hepatic encephalopathy (HE) during follow-up, which might offer potential biomarkers for the prognosis of TIPS.

## 2 Materials and methods

### 2.1 Patients and sample collection

We prospectively enrolled patients who underwent TIPS in our department from December 2019 to July 2020. Patients with any of the following characteristics were excluded: 1) no paired pre- and post-TIPS serum samples, 2) age <18 years, 3) missing clinical information, and 4) presence of diabetes or other metabolic diseases. Portal and peripheral blood samples were collected before and 24 h after the portosystemic shunt was developed. All blood samples were centrifuged at 3500 rpm for 10 min at 4 °C, and serum samples were collected. The serum samples were stored at −80 °C until analysis. The study protocol was predefined and adhered to the principles of the Helsinki Declaration II and was approved by the Ethics Committee of Shijitan Hospital [2019 (01)]. Written informed consent was obtained from all patients.

### 2.2 Chemicals and reagents

High-performance liquid chromatography-grade acetonitrile was obtained from Thermo Fisher Scientific (Waltham, MA, United States of America; A9554). Formic acid was purchased from Sigma–Aldrich (St. Louis, MO, United States ; 64186). Ultrapure water was produced by a Milli-Q water purification system (Millipore; Bedford, MA, United States).

### 2.3 Serum sample pretreatment

Before analysis, serum samples were thawed at 4°C. Then 50 μL of serum was added to a 1.5 mL EP tube, which contained 200 μL of cold extract solution (acetonitrile: methanol = 1:1). Mixed samples were put under vortex movement for 30 s and precipitated at −20°C for 30 min. After two centrifugation steps (12000 rpm, 4°C, 10 min), the supernatant was transferred to a clean tube for untargeted metabolomics analysis.

### 2.4 Untargeted metabolomics by UHPLC-QTOF/MS

Serum separation was performed using a UHPLC equipped with an ACQUITY UPLC HSS T3 C18 column (2.1 × 100 mm, 1.7 μm). Mobile phase A and B consisted of 0.1% (v/v) formic acid in H2O and acetonitrile, respectively. The elution gradient was set as follows: 1% B, 0–1.5 min; 1%–99% B, 1.0–20.0 min; 99% B, 20.0–25.0 min; 99%–1% B, 25.0–25.1 min; 1% B, 25.1–30.0 min. The column temperature was 30°C, the flow rate was 0.3 mL/min, and the injection volume was 3 μL for each run. The metabolomics profiling analysis was performed on a Xevo G2-XS Q-TOF mass system and the MassLynx v14.1 workstation (Waters; Milford, MA, United States). The source conditions in the positive or negative modes were set as follows: the capillary voltage was set at 3.0 kV for positive mode and 2.5 kV for negative mode; the sampling cone voltage was set at 40 V; the desolation gas flow rate was maintained at 600 L/h with a cone gas flow rate of 50 L/h; the desolation temperature was set at 350°C for positive mode and 500°C for negative mode; the source temperature was 100°C; and the TOF/MS full scan range was 50–1,000 Da. To ensure precise and stable scanning, leucine encephalin was used as a lock spray reference (m/z 556.2771 for positive mode and m/z 554.2615 for negative mode). Quality controls (QCs) were injected every 10 samples throughout the analytical run to provide a dataset for assessing repeatability.

### 2.5 Statistics

The raw data were packaged and imported into Progenesis QI v2.4 (Waters; Milford, MA, United States of America) for data processing, including baseline filtering, peak recognition, integration, retention time correction, peak alignment, peak picking, and normalization. Data normalization used unit variance scaling. Databases HMDB (https://www.hmdb.ca/) and LIPID MAPS (https://lipidmaps.org/) were applied for metabolite annotation, which was based on exact mass measurement (mass error <5 ppm), fragments of MS/MS, and isotope distribution obtained from UHPLC-QTOF/MS.

All statistical tests were performed with R v4.1 and the MetaboAnalyst 5.0 platform (https://www.metaboanalyst.ca). The coefficients of variation (CV) of QCs and principal component analysis were applied to monitor the stability and reproducibility of metabolomics datasets. A supervised model of orthogonal partial least squares discriminant analysis (OPLS-DA) was performed to maximize the distance between groups and identify significant metabolites according to their variable importance in the projection (VIP). Metabolites with VIP >1, *p* values <0.05, and fold-change ≥2 or ≤0.5 were considered significant differences and are shown in volcano plots. The correlations between significant metabolites and clinical parameters were conducted by Spearman’s correlation and are shown in hierarchical clustering heatmap. The clinical parameters included alanine aminotransferase (ALT), aspartate aminotransferase (AST), gamma-glutamyl transpeptidase (GGT), alkaline phosphatase (ALP), total protein (TP), albumin (ALB), total bilirubin (TBIL), direct bilirubin (DBIL), indirect bilirubin (IBIL), creatinine (CRE), activated partial thromboplastin time (APTT), prothrombin time (PT), international normalized ratio (INR), white blood cell count (WBC), hemoglobin (HGB), platelets (PLT), and portal pressure gradient (PPG). Metabolite set enrichment analysis (MSEA) was utilized to explore biologically meaningful patterns. Receiver operating characteristic curve (ROC) analysis calculates the predictive value of the metabolites.

## 3 Results

### 3.1 Baseline characteristics of patients

**FIGURE 1:**
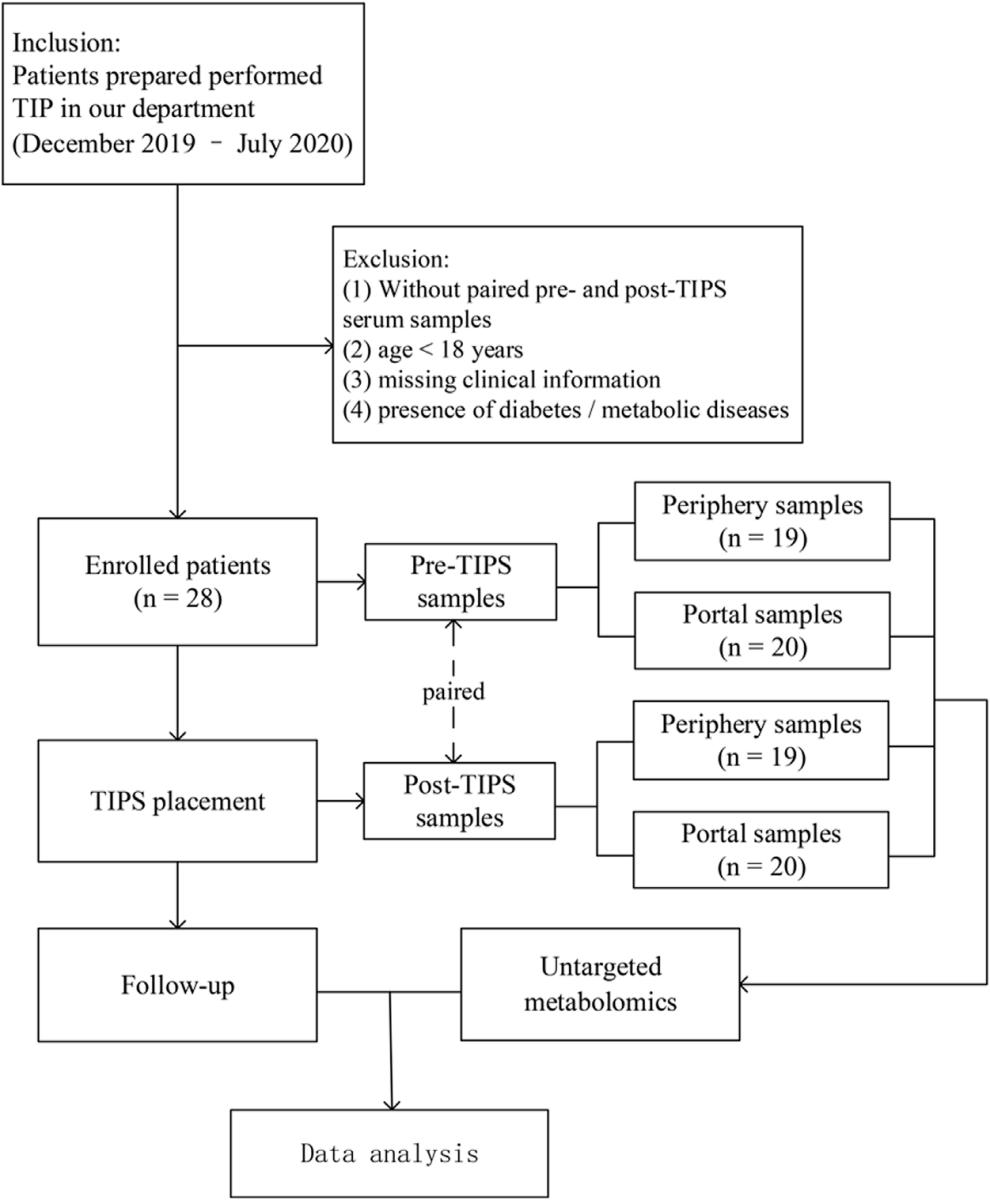
Design flow of the study.

A total of 28 patients were enrolled in this study, and 78 serum samples were collected from these patients, including 19 paired pre- and post-TIPS peripheral serum samples, and 20 paired pre- and post-TIPS portal serum samples (Figure 1). The baseline characteristics of the patients are summarized in Table 1.

**TABLE 1:** Baseline characteristics of patients.

Parameters	Number of patients (%)	Median (range)
Gender		
Male	54	
Female	46	
Age (years)		58 (28–79)
BMI		23.2 (16.0–37.0)
Cause of portal hypertension		
Alcoholic	29	
Virus	21	
Budd-Chiari syndrome	11	
Idiopathic portal hypertension	11	
Autoimmune liver disease	7	
Others	21	
Ascites		
Absent	29	
Mild	14	
Severe	57	
Esophagogastric varices		
Mild	15	
Severe	85	
Variceal bleeding		
Absent	21	
Present	79	
Cancer		
Absent	71	
Present	29	
Stent diameter		
8 mm	82	
10 mm	18	
Child-Pugh score		7 (5–11)
Child-Pugh category		
A	32	
B	54	
C	14	
MELD score		12.5 (4.7–26.3)
PPG (mmHg)		26 (5–37)
Bilirubin (μmol/L)		26.0 (2.5–273.3)
Creatinine (μmol/L)		60 (36–224)
INR		1.3 (1.0–1.9)
Albumin (g/L)		33.2 (24.7–56.1)

Abbreviations: BMI, body mass index; MELD, model for end-of-stage liver disease; PPG, portal pressure gradient; INR, international normalized ratio.

### 3.2 Analytical method of assessing metabolomics

**FIGURE 2:**
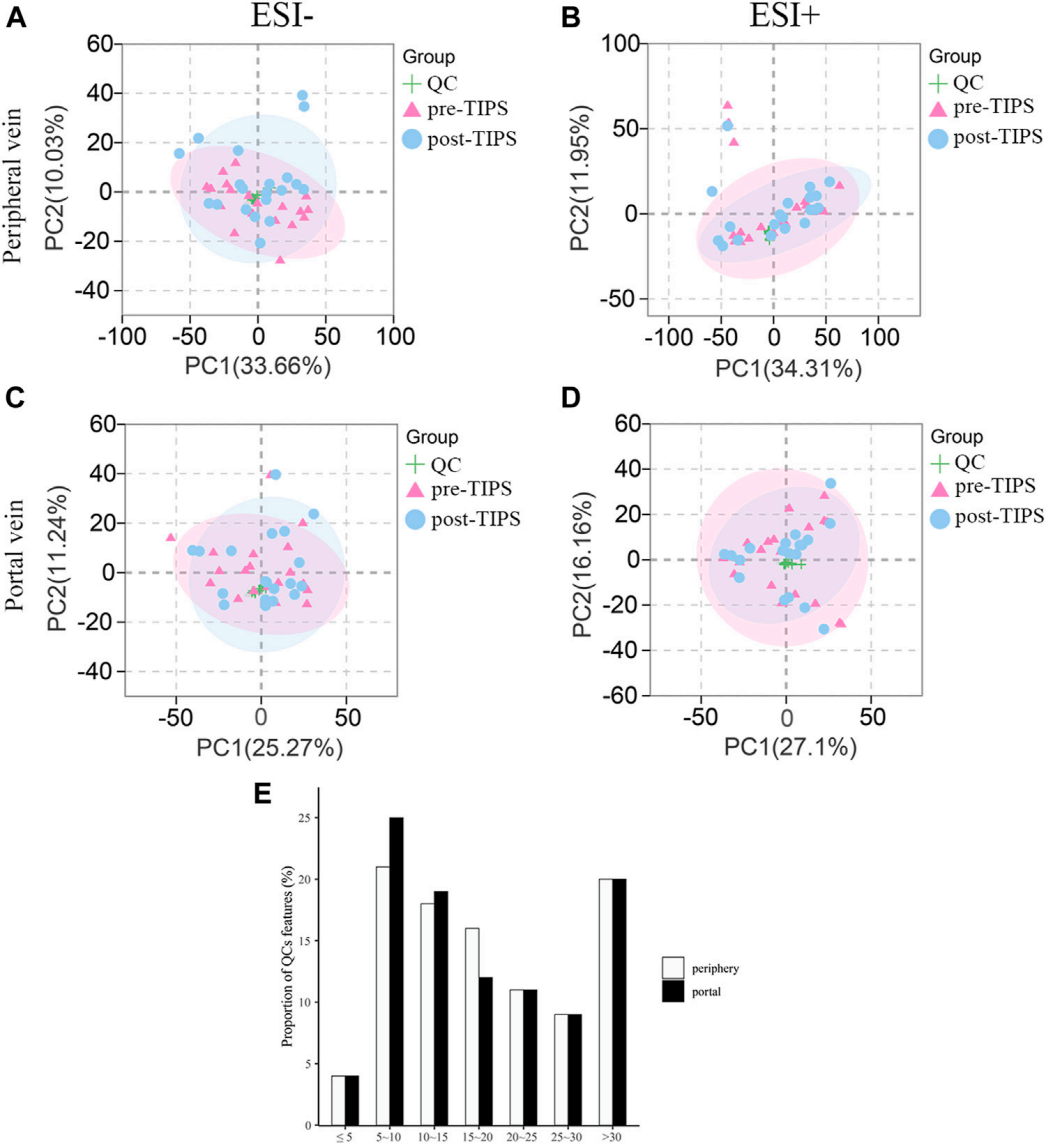
Principal component analysis (PCA) score plots for all samples and coefficients of variation distribution of quality control (QC) samples. QC samples cluster tightly together in both ESI- and ESI + mode of peripheral **(A, B)** and portal serum **(C, D)**. **(E)** Coefficients of variation distribution of QC samples in the combinational dataset of ESI+ and ESI- modes from the peripheral and portal serum. ESI-: negative electrospray ionization; ESI+: positive electrospray ionization.

The CV of positive/negative ions in QC samples was calculated to evaluate the method’s stability. 25% and 29% of ions in the peripheral and portal QC samples exhibited CV < 15%, and 79% and 80% of ions in the peripheral and portal QC samples displayed CV < 30% (Figure 2E). These results suggested that the UHPLC-QTOF/MS system showed relatively high reproducibility and stability. Furthermore, in the principal component analysis score plots of negative electrospray ionization (ESI-) and positive electrospray ionization (ESI+) models of all serum samples, QC samples cluster tightly together, indicating that the data quality is stable and reliable (Figures 2A–D).

### 3.3 Metabolites in the peripheral and portal serum significantly changed early after TIPS

**FIGURE 3:**
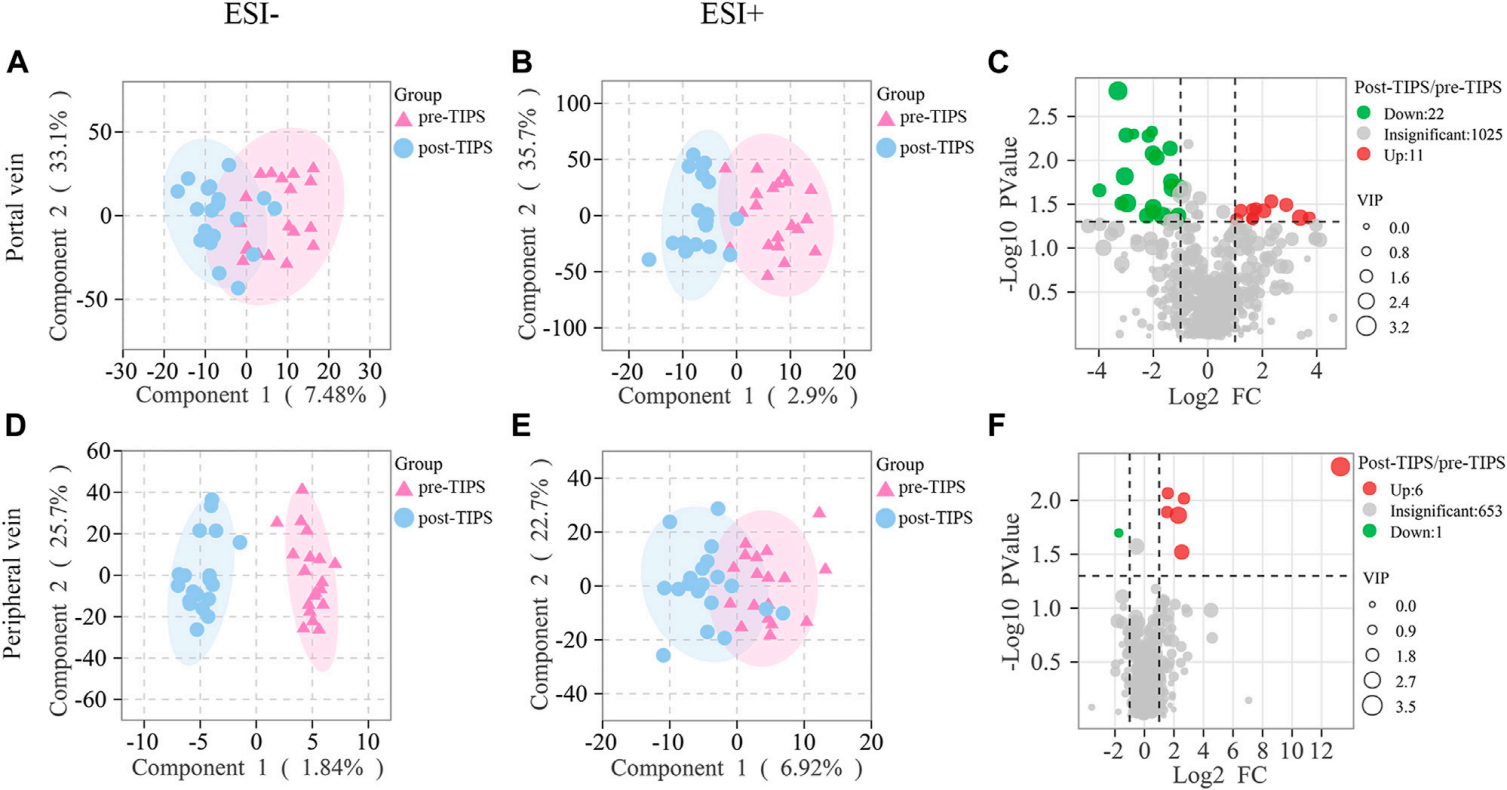
Metabolites in peripheral and portal serum significantly changed early after TIPS. Both ESI- **(A)** and ESI+ **(B)** models of orthogonal partial least squares discriminant analysis (OPLS-DA) of portal metabolites were separated. Both ESI- **(D)** and ESI+ **(E)** models of peripheral metabolites were separated. Volcano plots showed significant metabolic changes in portal **(C)** and peripheral **(F)** with *p*-value <1, variable importance in the projection (VIP) > 1, and fold change ≥2 or ≤0.5.

In ESI- (Figures 3A,D) and ESI+ (Figures 3B,E) OPLS-DA score plots, pre- and post-TIPS metabolites were separated. Metabolites with VIP >1, *p*-value <0.05, and FC ≥ 2 or ≤0.5 were identified as significantly changed after TIPS. Volcano plots showed the significantly changed metabolites in the portal (Figure 3C) and peripheral serum (Figure 3F), including 11 increased metabolites and 22 decreased metabolites in portal serum and 6 increased metabolites and 1 decreased metabolite in peripheral serum. When the false discovery rate <0.05 criterion was applied, only PC(O-20:0/O-1:0) in portal serum and 5-Pentacosyl-1,3-benzenediol in periphery serum were significantly different between patients with and without post-TIPS HE. Portal serum may offer more metabolic information as more significantly altered metabolites were observed in portal serum. Most of the above significantly altered metabolites belong to lipids, amino acids, and peptides.

### 3.4 Correlations between significantly altered metabolites and clinical parameters

**FIGURE 4:**
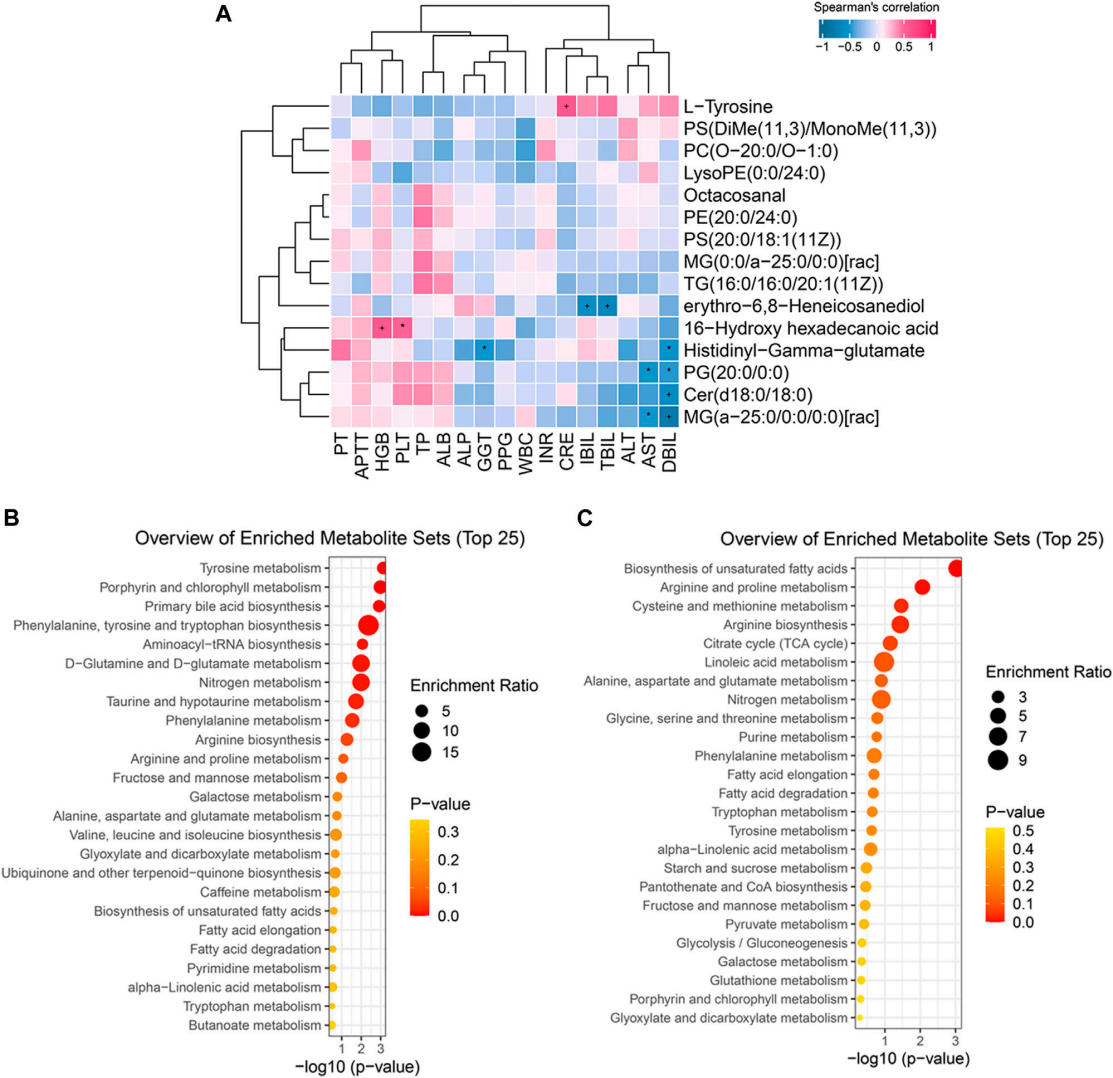
Biologically meaningful patterns of significant metabolites. **(A)** Some metabolic changes were significantly associated with liver function parameters. **(B)** The enriched metabolites sets in increasing metabolic changes. **(C)** The enriched metabolites sets in decreasing metabolic changes.

The correlations between significantly altered metabolites and clinical parameters were evaluated by Spearman’s correlation analysis and are shown in hierarchical clustering heatmaps (Figure 4A): some metabolites were significantly related to liver function parameters, such as AST, GGT, CRE, TBIL, DBIL, and IBIL (*p* < 0·05). Depleted lipid metabolites erythro-6,8-Heneicosanediol, PG (20:0/0:0), Cer(d18:0/18:0), and MG (a-25:0/0:0/0:0)[rac] were negatively correlated with TBIL, IBIL, DBIL, and AST, respectively. These results indicated that the decrease in lipid metabolites might be partly ascribed to the deterioration of liver function. Additionally, decreased 16-hydroxy hexadecanoic acid was positively related to HGB and PLT, suggesting that altering blood components might affect the change in metabolites after TIPS.

### 3.5 Metabolite set enrichment analysis (MSEA) of the significant metabolites

To further clarify the function of the significant metabolic changes, we performed MSEA on significant lipid metabolites, amino acids, and peptides. The results demonstrated that elevated metabolites were mainly involved in amino acid metabolism, including tyrosine metabolism, phenylalanine, tyrosine and tryptophan biosynthesis, D−glutamine and D−glutamate metabolism, phenylalanine metabolism, arginine biosynthesis, arginine and proline metabolism, alanine, aspartate and glutamate metabolism, and valine, leucine and isoleucine biosynthesis (Figure 4B). Similarly, depleted metabolites enriched in several amino acid metabolisms, such as arginine and proline metabolism, cysteine and methionine metabolism, arginine biosynthesis, alanine, aspartate and glutamate metabolism, glycine, serine and threonine metabolism, etc (Figure 4C). These results suggest that post-TIPS metabolism may switch to amino acid metabolism, generating more protein to promote the body’s recovery.

### 3.6 Post-TIPS metabolic changes might be potential predictive biomarkers of adverse events

**FIGURE 5:**
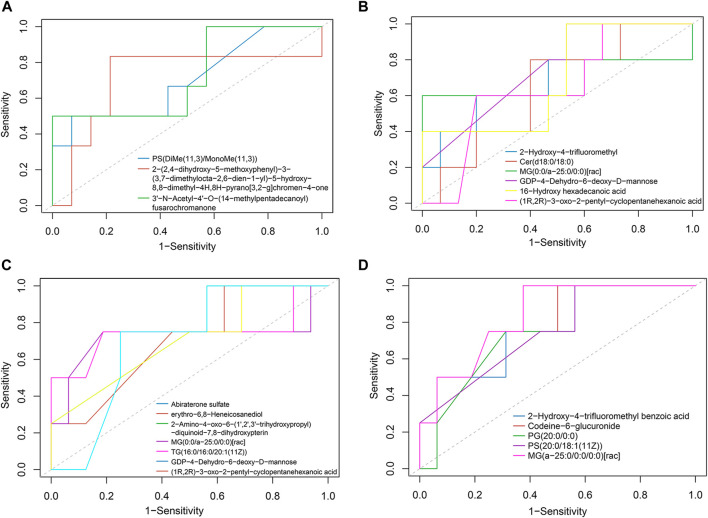
Potential biomarker of adverse events after TIPS. **(A)** Increased portal metabolites had good predictive value in liver function decline (AUC >0.7). **(B)** Decreased portal metabolites with good predictive value in liver function decline (AUC >0.7). **(C)** Depleted portal metabolites performed a good distinctive value in HE (0.7< AUC <0.75). **(D)** Five decreased portal metabolites had better predictive value in post-TIPS HE (AUC ≥0.75).

To explore the role of these metabolic changes in clinical practice, we calculated the predictive value of the metabolic changes in adverse events after TIPS, such as liver function decline and HE. Patients with Child-Pugh scores that increased over 2 points (∆CP ≥ 2) within 1 year were defined as having significant liver function decline. Nine of 28 patients had considerable liver function damage 1 year after TIPS. ROC analysis suggested 3 increased metabolites (Figure 5A) and 6 decreased metabolites (Figure 5B) in portal serum with an area under the curve (AUC) > 0.7. Their AUCs are shown in Table 2. These metabolites would be potential predictive biomarkers of liver function decline after TIPS. Additionally, HE was diagnosed according to the West Haven criteria (Conn et al., 1977), and 6/28 patients developed post-TIPS HE within 1 year. ROC analysis of HE indicated 12 depleted metabolites in portal serum with AUC >0.7 (Figures 5C,D), and 5 of them produced a better classification performance with an AUC >0.75 (Figure 5D). Their AUCs and 95% confidence intervals are shown in Table 3. However, only the AUC of GDP-4-Dehydro-6-deoxy-D-mannose (Table 2) and MG (a-25:0/0:0/0:0) [rac] (Table 3) with *p* < 0.05, so these metabolites should be paid more attention in the future research.

**TABLE 2:** Receiver operating characteristic curve analysis of liver function decline.

Metabolites	AUC	95% CI	*p*-value
3′-N-Acetyl-4′-O-(14-methylpentadecanoyl)fusarochromanone	0.726	0.452–1.000	0.0648
2-(2,4-dihydroxy-5-methoxyphenyl)-3-(3,7-dimethylocta-2,6-dien-1-yl)-5-hydroxy-8,8-dimethyl-4H,8H-pyrano [3,2-g]chromen-4-one	0.714	0.400–1.000	0.0764
PS[DiMe(11,3)/MonoMe(11,3)]	0.702	0.426–0.979	0.0839
2-Hydroxy-4-trifluoromethyl benzoic acid	0.708	0.389–1.000	0.0804
Cer(d18:0/18:0)	0.738	0.483–0.993	0.0547
MG (0:0/a-25:0/0:0)[rac]	0.702	0.367–1.00	0.086
GDP-4-Dehydro-6-deoxy-D-mannose	0.774	0.591–0.956	0.0237
(1R,2R)-3-oxo-2-pentyl-cyclopentanehexanoic acid	0.732	0.474–0.990	0.0579
16-Hydroxy hexadecanoic acid	0.702	0.449–0.956	0.0893

Abbreviations: AUC, area under the curve; 95% CI, 95% confidence interval.

**TABLE 3:** Receiver operating characteristic curve analysis of hepatic encephalopathy.

Metabolites	AUC	95% CI	*p*-value
Abiraterone sulfate	0.734	0.478–0.991	0.0716
erythro-6,8-Heneicosanediol	0.703	0.418–0.988	0.1138
2-Amino-4-oxo-6-(1′,2′,3′-trihydroxypropyl)-diquinoid-7,8-dihydroxypterin	0.734	0.478–0.991	0.0716
MG (0:0/a-25:0/0:0)[rac]	0.719	0.281–1.000	0.1007
TG (16:0/16:0/20:1 (11Z))	0.742	0.327–1.000	0.0781
GDP-4-Dehydro-6-deoxy-D-mannose	0.703	0.402–1.000	0.1035
(1R,2R)-3-oxo-2-pentyl-cyclopentanehexanoic acid	0.703	0.451–0.955	0.1179
2-Hydroxy-4-trifluoromethyl benzoic acid	0.766	0.486–1.000	0.0594
Codeine-6-glucuronide	0.766	0.531–1.000	0.0511
PG (20:0/0:0)	0.750	0.495–1.000	0.0689
PS(20:0/18:1 (11Z))	0.750	0.493–1.000	0.0623
MG (a-25:0/0:0/0:0)[rac]	0.836	0.632–1.000	0.0236

Abbreviations: AUC, area under the curve; 95% CI, 95% confidence interval.

## 4 Discussion

TIPS is a common and effective treatment for portal hypertension (Tripathi et al., 2020; Horhat et al., 2021). However, the effect of TIPS on body components is unclear, which might influence the prognosis of TIPS. Few studies have explored body component changes following TIPS, and it was found that TIPS might improve sarcopenia by increasing muscle and fat mass (Dasarathy et al., 2011; Thomsen et al., 2012; Artru et al., 2020). However, little is known regarding the metabolomic change after TIPS. This study showed that some metabolites significantly changed early after TIPS, especially peptides, amino acids, and lipid metabolites. Some metabolic changes were significantly associated with the alternation of liver function early after TIPS. Notably, some early metabolic changes had moderate predictive value in liver function decline and post-TIPS HE after TIPS.

Over 50% of decompensated cirrhosis cases are accompanied by sarcopenia (European Association for the Study of the Liver, 2019; Cruz-Jentoft et al., 2019; Traub et al., 2021). Sarcopenia has been demonstrated to be an independent risk factor for mortality in cirrhosis patients (Ebadi et al., 2019; Tantai et al., 2022). Several studies have indicated that TIPS could improve sarcopenia by increasing muscle and fat mass (Artru et al., 2020; Liu et al., 2022). Similarly, this study found that metabolites, especially peptides, amino acids, and lipid metabolites significantly changed early after TIPS. Of note, the significantly increased metabolites included peptides, amino acids, and lipid metabolites, but the most significantly depleted metabolites were lipid metabolites. In a previous study, it was also observed that the subcutaneous fat surface increased, but the visceral fat surface decreased after TIPS (Artru et al., 2020). Thus, the post-TIPS alteration of lipid metabolites is complicated. Furthermore, metabolite set enrichment analysis showed that the increased metabolites were mainly enriched in amino acid metabolism, indicating that elevated amino acids might be more critical in metabolite changes after TIPS. Sarcopenic obesity and myosteatosis are frequently observed in patients with cirrhosis (Montano-Loza et al., 2016; Ahn et al., 2021). Therefore, the complicated lipid metabolism might result from amino acid metabolism. Additionally, more significant metabolites appeared in portal serum, demonstrating that portal metabolites might offer more insight into metabolic changes after TIPS. Hence, we focused on portal metabolic changes in subsequent analysis.

The correlation analysis showed that some metabolic changes were significantly related to the alteration in liver function after TIPS. For example, depletion of erythro-6,8-Heneicosanediol, PG (20:0/0:0), and Cer(d18:0/18:0) was negatively correlated with changes of TBIL, IBIL, and DBIL, respectively. A high concentration of bilirubin implies hepatocellular injury or biliary obstruction in most settings (Kwo et al., 2017). Generally, impaired hepatocytes could not produce more lipid metabolites through *de novo* lipogenesis (Badmus et al., 2022). Furthermore, circulating fatty acids taken up by liver could not be disposed of because fatty acid oxidation and lipids export would reduce during liver dysfunction, leading to circulating lipid metabolites decrease and liver lipids accumulation (Ipsen et al., 2018). A large number of accumulated lipids produce hepatotoxicity, further impairing liver function and forming a vicious circle (Ipsen et al., 2018; Badmus et al., 2022). The above-mention hepatic lipid metabolism mechanism might be the reason that liver dysfunction might impact lipid metabolite changes after TIPS.

Additionally, apart from the liver function parameters, the alteration of blood components, such as HGB and PLT, also affects the change in metabolites after TIPS. No significant association with other factors, such as PPG and inflammation, was found in our study. However, most metabolites were not significantly associated with clinical parameters. Thus, other factors might also affect the change in metabolites. Further research is needed to explore the reason for the metabolic changes after TIPS.

Importantly, our study demonstrated that early metabolite changes might be involved in adverse events after TIPS. ROC analysis found 3 increased and 6 depleted metabolites in portal serum had moderate predictive value in liver function decline, which mainly belonged to lipid metabolites, such as PS(DiMe(11,3)/MonoMe(11,3)), Cer(d18:0/18:0), and MG (0:0/a-25:0/0:0)[rac]. These results further confirmed the vital value of lipid metabolites in post-TIPS outcomes. Similarly, 5 depleted metabolites in portal serum showed moderate performance in the prediction of post-TIPS HE, including 2-Hydroxy-4-trifluoromethyl benzoic acid, Codeine-6-glucuronide, PG (20:0/0:0), PS(20:0/18:1 (11Z)), and MG (a-25:0/0:0/0:0)[rac]. However, only the AUC of GDP-4-Dehydro-6-deoxy-D-mannose and MG (a-25:0/0:0/0:0) [rac] have statistical significance. Thus, GDP-4-Dehydro-6-deoxy-D-mannose (Table 2) and MG (a-25:0/0:0/0:0) [rac] should be paid more attention to in future research.

To the best of our knowledge, little research focuses on post-TIPS metabolic changes, so this study is explorative. However, our study is limited by the small sample size and the absence of strict dietary restrictions for patients during follow-up. To minimize the impact of our limitations, we evaluated the metabolic changes in the peripheral and portal serum, applied conservative statistical criteria, and combine univariate and multivariate analysis to identify significant changes. However, some results were still insignificant because of the small sample size, such as several metabolites with AUC >0.7 but *p* > 0.05. Thus, further in-depth studies with larger sample sizes and multiple centers are warranted to validate the changes after TIPS and validate the predictive value of these metabolites in clinical practice.

In conclusion, the peripheral and portal metabolites significantly changed after TIPS. More significant metabolic changes were observed in portal serum, so the metabolites in portal serum might offer more insight into metabolic alteration after TIPS. Some metabolic changes might be ascribed to a decline in liver function. Importantly, 9 portal metabolites have good predictive value in post-TIPS liver function decline, and 12 portal metabolites have a moderate classification performance in HE, which might offer a reference for biomarker research.

## Data Availability

The datasets presented in this study can be found in MetaboLights using accession number MTBLS7800 (https://www.ebi.ac.uk/metabolights/editor/ www.ebi.ac.uk/metabolights/MTBLS7800).

## References

[B1] Ahn H. Kim D. W. Ko Y. Ha J. Shin Y. B. Lee J. (2021). Updated systematic review and meta-analysis on diagnostic issues and the prognostic impact of myosteatosis: A new paradigm beyond sarcopenia. Ageing Res. Rev. 70, 101398. 10.1016/j.arr.2021.101398 34214642

[B2] Artru F. Miquet X. Azahaf M. Labreuche J. Ntandja Wandji L. C. Sergent G. (2020). Consequences of TIPSS placement on the body composition of patients with cirrhosis and severe portal hypertension: A large retrospective CT-based surveillance. Aliment. Pharmacol. Ther. 52 (9), 1516–1526. 10.1111/apt.16080 32931618

[B3] Badmus O. O. Hillhouse S. A. Anderson C. D. Hinds T. D. Stec D. E. (2022). Molecular mechanisms of metabolic associated fatty liver disease (MAFLD): Functional analysis of lipid metabolism pathways. Clin. Sci. 136 (18), 1347–1366. 10.1042/cs20220572 PMC9508552 36148775

[B4] Bureau C. Thabut D. Oberti F. Dharancy S. Carbonell N. Bouvier A. (2017). Transjugular intrahepatic portosystemic shunts with covered stents increase transplant-free survival of patients with cirrhosis and recurrent ascites. Gastroenterology 152 (1), 157–163. 10.1053/j.gastro.2016.09.016 27663604

[B5] Chen C. J. Lee D. Y. Yu J. Lin Y. N. Lin T. M. (2022). Recent advances in LC-MS-based metabolomics for clinical biomarker discovery. Mass Spectrom. Rev., e21785. 10.1002/mas.21785 35645144

[B6] Claria J. Curto A. Moreau R. Colsch B. Lopez-Vicario C. Lozano J. J. (2021). Untargeted lipidomics uncovers lipid signatures that distinguish severe from moderate forms of acutely decompensated cirrhosis. J. Hepatol. 75 (5), 1116–1127. 10.1016/j.jhep.2021.06.043 34245803

[B7] Conn H. O. Leevy C. M. Vlahcevic Z. R. Rodgers J. B. Maddrey W. C. Seeff L. (1977). Comparison of lactulose and neomycin in the treatment of chronic portal-systemic encephalopathy. Gastroenterology 72 (4), 573–583. 10.1016/s0016-5085(77)80135-2 14049

[B8] Cruz-Jentoft A. J. Bahat G. Bauer J. Boirie Y. Bruyère O. Cederholm T. (2019). Sarcopenia: Revised European consensus on definition and diagnosis. Age Ageing 48 (4), 601. 10.1093/ageing/afz046 PMC6593317 31081853

[B9] Dasarathy J. Alkhouri N. Dasarathy S. (2011). Changes in body composition after transjugular intrahepatic portosystemic stent in cirrhosis: A critical review of literature. Liver Int. 31 (9), 1250–1258. 10.1111/j.1478-3231.2011.02498.x 21745273

[B10] Ebadi M. Bhanji R. A. Mazurak V. C. Montano-Loza A. J. (2019). Sarcopenia in cirrhosis: From pathogenesis to interventions. J. Gastroenterol. 54 (10), 845–859. 10.1007/s00535-019-01605-6 31392488 PMC6759678

[B11] European Association for the Study of the Liver (2019). EASL Clinical Practice Guidelines on nutrition in chronic liver disease. J. Hepatol. 70 (1), 172–193. 10.1016/j.jhep.2018.06.024 30144956 PMC6657019

[B12] Fan Y. Li Y. Chen Y. Zhao Y. J. Liu L. W. Li J. (2016). Comprehensive metabolomic characterization of coronary artery diseases. J. Am. Coll. Cardiol. 68 (12), 1281–1293. 10.1016/j.jacc.2016.06.044 27634119

[B13] García-Pagán J. C. Caca K. Bureau C. Laleman W. Appenrodt B. Luca A. (2010). Early use of TIPS in patients with cirrhosis and variceal bleeding. N. Engl. J. Med. 362 (25), 2370–2379. 10.1056/NEJMoa0910102 20573925

[B14] Horhat A. Bureau C. Thabut D. Rudler M. (2021). Transjugular intrahepatic portosystemic shunt in patients with cirrhosis: Indications and posttransjugular intrahepatic portosystemic shunt complications in 2020. United Eur. Gastroenterol. J. 9 (2), 203–208. 10.1177/2050640620952637 PMC8259430 32819214

[B15] Ipsen D. H. Lykkesfeldt J. Tveden-Nyborg P. (2018). Molecular mechanisms of hepatic lipid accumulation in non-alcoholic fatty liver disease. Cell Mol. Life Sci. 75 (18), 3313–3327. 10.1007/s00018-018-2860-6 29936596 PMC6105174

[B16] Jahangiri Y. Pathak P. Tomozawa Y. Li L. Schlansky B. L. Farsad K. (2019). Muscle gain after transjugular intrahepatic portosystemic shunt creation: Time course and prognostic implications for survival in cirrhosis. J. Vasc. Interv. Radiol. 30 (6), 866–872. 10.1016/j.jvir.2019.01.005 31053265

[B17] Judge A. Dodd M. S. (2020). Metabolism. Essays Biochem. 64 (4), 607–647. 10.1042/ebc20190041 32830223 PMC7545035

[B18] Kwo P. Y. Cohen S. M. Lim J. K. (2017). ACG clinical guideline: Evaluation of abnormal liver chemistries. Am. J. Gastroenterol. 112 (1), 18–35. 10.1038/ajg.2016.517 27995906

[B19] Liu J. Ma J. Yang C. Chen M. Shi Q. Zhou C. (2022). Sarcopenia in patients with cirrhosis after transjugular intrahepatic portosystemic shunt placement. Radiology 303, 711–719. 10.1148/radiol.211172 35289658

[B20] Luo P. Yin P. Hua R. Tan Y. Li Z. Qiu G. (2018). A Large-scale, multicenter serum metabolite biomarker identification study for the early detection of hepatocellular carcinoma. Hepatology 67 (2), 662–675. 10.1002/hep.29561 28960374 PMC6680350

[B21] Miyoshi H. Moriya K. Tsutsumi T. Shinzawa S. Fujie H. Shintani Y. (2011). Pathogenesis of lipid metabolism disorder in hepatitis C: Polyunsaturated fatty acids counteract lipid alterations induced by the core protein. J. Hepatol. 54 (3), 432–438. 10.1016/j.jhep.2010.07.039 21093950

[B22] Montano-Loza A. J. Angulo P. Meza-Junco J. Prado C. M. Sawyer M. B. Beaumont C. (2016). Sarcopenic obesity and myosteatosis are associated with higher mortality in patients with cirrhosis. J. Cachexia Sarcopenia Muscle 7 (2), 126–135. 10.1002/jcsm.12039 27493866 PMC4864157

[B23] Plauth M. Schutz T. Buckendahl D. P. Kreymann G. Pirlich M. Grungreiff S. (2004). Weight gain after transjugular intrahepatic portosystemic shunt is associated with improvement in body composition in malnourished patients with cirrhosis and hypermetabolism. J. Hepatol. 40 (2), 228–233. 10.1016/j.jhep.2003.10.011 14739092

[B24] Ponziani F. R. Picca A. Marzetti E. Calvani R. Conta G. Del Chierico F. (2021). Characterization of the gut-liver-muscle axis in cirrhotic patients with sarcopenia. Liver Int. 41 (6), 1320–1334. 10.1111/liv.14876 33713524

[B25] Simonetto D. A. Liu M. Kamath P. S. (2019). Portal hypertension and related complications: Diagnosis and management. Mayo Clin. Proc. 94 (4), 714–726. 10.1016/j.mayocp.2018.12.020 30947834

[B26] Stolzenberg-Solomon R. Derkach A. Moore S. Weinstein S. J. Albanes D. Sampson J. (2020). Associations between metabolites and pancreatic cancer risk in a large prospective epidemiological study. Gut 69 (11), 2008–2015. 10.1136/gutjnl-2019-319811 32060129 PMC7980697

[B27] Tandon P. Montano-Loza A. J. Lai J. C. Dasarathy S. Merli M. (2021). Sarcopenia and frailty in decompensated cirrhosis. J. Hepatol. 75 (1), S147-s162. 10.1016/j.jhep.2021.01.025 34039486 PMC9125684

[B28] Tantai X. Liu Y. Yeo Y. H. Praktiknjo M. Mauro E. Hamaguchi Y. (2022). Effect of sarcopenia on survival in patients with cirrhosis: A meta-analysis. J. Hepatol. 76 (3), 588–599. 10.1016/j.jhep.2021.11.006 34785325

[B29] Thomsen K. L. Sandahl T. D. Holland-Fischer P. Jessen N. Frystyk J. Flyvbjerg A. (2012). Changes in adipokines after transjugular intrahepatic porto-systemic shunt indicate an anabolic shift in metabolism. Clin. Nutr. 31 (6), 940–945. 10.1016/j.clnu.2012.04.001 22541535

[B30] Traub J. Reiss L. Aliwa B. Stadlbauer V. (2021). Malnutrition in patients with liver cirrhosis. Nutrients 13 (2), 540. 10.3390/nu13020540 33562292 PMC7915767

[B31] Tripathi D. Stanley A. J. Hayes P. C. Travis S. Armstrong M. J. Tsochatzis E. A. (2020). Transjugular intrahepatic portosystemic stent-shunt in the management of portal hypertension. Gut 69 (7), 1173–1192. 10.1136/gutjnl-2019-320221 32114503 PMC7306985

[B32] Trotter J. F. Suhocki P. V. Rockey D. C. (1998). Transjugular intrahepatic portosystemic shunt (TIPS) in patients with refractory ascites: Effect on body weight and child-pugh score. Am. J. Gastroenterol. 93 (10), 1891–1894. 10.1111/j.1572-0241.1998.00544.x 9772050

[B33] Tsochatzis E. A. Bosch J. Burroughs A. K. (2014). Liver cirrhosis. Lancet 383 (9930), 1749–1761. 10.1016/s0140-6736(14)60121-5 24480518

[B34] Wang X. Zhang A. Sun H. (2013). Power of metabolomics in diagnosis and biomarker discovery of hepatocellular carcinoma. Hepatology 57 (5), 2072–2077. 10.1002/hep.26130 23150189

